# Cofeeding tolerance in chimpanzees depends on group composition: A longitudinal study across four communities

**DOI:** 10.1016/j.isci.2021.102453

**Published:** 2021-04-29

**Authors:** Sarah E. DeTroy, Cody T. Ross, Katherine A. Cronin, Edwin J.C. van Leeuwen, Daniel B.M. Haun

## Main text

(iScience *24*, 102175-1–102175-13; March 19, 2021)

Due to an editorial and production oversight, a PDF of the uncopyedited manuscript text was mistakenly uploaded to the final published version of the article instead of the supplemental information PDF. This has since been corrected online. The editorial and production teams apologize for this error.

