# Supplementary material for: Cofeeding tolerance in chimpanzees depends on group composition: a longitudinal study across four communities
Source: iScience. 2021 Feb 12;24(3):102175. doi: 10.1016/j.isci.2021.102175 (PMC7940988; doi:10.1016/j.isci.2021.102175)
Supplement: Document S1. Transparent methods, Figures S1–S13, and Tables S1–S4 [file mmc1.pdf]

iScience, Volume *24*

Supplemental information

Cofeeding tolerance in chimpanzees depends on group composition: a longitudinal study across four communities

Sarah E. DeTroy, Cody T. Ross, Katherine A. Cronin, Edwin J.C. van Leeuwen, and Daniel B.M. Haun

Cofeeding Tolerance in Chimpanzees Depends on Group Composition: A Longitudinal Study across Four Communities

Sarah E. DeTroy[1,2,8*], Cody T. Ross[1], Katherine A. Cronin[3,4], Edwin J. C. van Leeuwen[1,5,6,7], Daniel B. M. Haun[1,2,7]

1 Max Planck Institute for Evolutionary Anthropology, Deutscher Platz 6, 04103 Leipzig, Germany
2 Department for Early Child Development and Culture, Faculty of Education, Leipzig University, Jahnallee 59, 04109 Leipzig, Germany
3 Animal Welfare Science Program, Lincoln Park Zoo, 2001 N Clark St, Chicago, IL 60614, United States of America
4 Committee on Evolutionary Biology, University of Chicago, 1025 E. 57th Street Chicago, IL 60637, United States of America
5 Behavioural Ecology and Ecophysiology Group, Department of Biology, University of Antwerp, Universiteitsplein 1, 2610 Antwerp, Belgium
6 Centre for Research and Conservation, Royal Zoological Society of Antwerp, Koningin Astridplein 20-26, 2018 Antwerp, Belgium
7 Senior Authors
8 Lead Contact

*Correspondence: sarah_detroy@eva.mpg.de

Summary

Social tolerance is generally treated as a stable, species-specific characteristic. Recent research, however, has questioned this position and emphasized the importance of intraspecific variation. We investigate the temporal stability of social tolerance in four groups of sanctuary-housed chimpanzees over eight years using a commonly employed measure: experimental cofeeding tolerance. We then draw on longitudinal data on the demographic composition of each group to identify the factors associated with cofeeding tolerance. We find appreciable levels of variation in cofeeding tolerance across both groups and years that correspond closely to changes in group-level demographic composition. For example, cofeeding tolerance is lower when there are many females with young infants. These results suggest that social tolerance may be a 'responding trait' of chimpanzee sociality, reflecting individual-level behavioral responses to social changes. Additional, experimental research is needed to better model the causal drivers of social tolerance within and among species.

Introduction

Successful group living requires individuals to routinely interact in a relaxed and non-antagonistic manner. Interaction styles in which antagonism is rare are often referred to as *socially tolerant*. Humans are assumed to be characterized by unusually high levels of such social tolerance (Cieri et al., 2014; Burkart et al., 2009; Fuentes, 2004; Pisor and Surbeck, 2019), as we are capable of living in large numbers and in close proximity with one another, as well as cooperating on a daily basis with complete strangers (Chudek and Henrich, 2011; Richerson et al., 2016). This social tolerance is hypothesized to have played a key role in the subsequent evolution of our supposedly unique expressions of prosociality, altruism, cooperation, and social learning (Hare, 2017; Cieri et al., 2014; Fuentes, 2004; Fehr and Fischbacher, 2003).

Social tolerance levels have been described in many different socially living species: e.g., voles (McShea, 1990; Lee et al., 2019), domestic chickens (D'Eath and Keeling, 2003), mole-rats (Ganem and Bennett, 2004), swallows

(Dardenne et al., 2013), crows (Miller et al., 2014), dogs (Bonanni et al., 2017; Hare, 2017), foxes (Hare, 2017), and dolphins (Wild et al., 2020). Researchers have investigated the relationship between social tolerance and group size (larger groups are associated with higher social tolerance (D'Eath and Keeling, 2003; Dardenne et al., 2013)), social learning (social tolerance enables social learning (Wild et al., 2020; Miller et al., 2014; Forss et al., 2016)), and domestication (domesticated species display higher levels of social tolerance than their wild counterparts (Bradshaw, 2016; Bonanni et al., 2017; Hare, 2017; Hare et al., 2012)). Social tolerance has frequently been utilized to characterize and compare entire species and subspecies: for example, the supposedly tolerant social mole rats and intolerant solitary mole rats (Ganem and Bennett, 2004), seasonally tolerant meadow voles and consistently intolerant prairie voles (Lee et al., 2019), tolerant domesticated foxes and intolerant wild foxes (Hare, 2017), or tolerant dogs and intolerant wolves (Hare et al., 2012).

This comparative approach is especially common in studies of primates, where the construct of social tolerance has played a central role in describing and differentiating the social behavior of related species. Macaque species are organized in social grades according to their described level of social tolerance (Thierry, 2007; Balasubramaniam et al., 2018), Sumatran orangutans are reported to be more tolerant than Bornean orangutans (Forss et al., 2016), redfronted lemurs more tolerant than ringtailed lemurs (Fichtel et al., 2018), and bonobos more tolerant than chimpanzees (Clay and de Waal, 2013; Hare et al., 2012; Tan and Hare, 2013), but see (Jaeggi et al., 2010; Cronin et al., 2015) for conflicting findings. These species-level assumptions have also been advanced to explain and predict interspecific variation in behaviors such as cooperation (Petit et al., 1992; Hare et al., 2007; Cronin, 2017), prosociality (Burkart and van Schaik, 2013; Burkart et al., 2014; Fruth and Hohmann, 2018; Cronin, 2012), and social learning (van Schaik, 2003; Schuppli et al., 2017; van Schaik et al., 1999),

Definitions and operationalizations of social tolerance among primates (and beyond) vary widely, encompassing measures as diverse as post-conflict reconciliation (Duboscq et al., 2013), grooming behavior (Balasubramaniam et al., 2018), and counter-aggression (Balasubramaniam et al., 2012). While there is, to date, no unifying methodological framework for the study of social tolerance, it is often operationalized either as an assessment of the social structure of a group or the expression of specific inter-individual behaviors (see DeTroy et al., Manuscript submitted for publication).

One of the most commonly used measures of socially tolerant behavior is *cofeeding tolerance*, both dyadically (e.g., Amici et al., 2012; Melis et al., 2006) and at a group-level (e.g., de Waal, 1986; Calcutt et al., 2014; Cronin et al., 2015; Fichtel et al., 2018). In these contexts, social tolerance has been defined as "the probability that individuals will be in proximity to conspecifics around valuable resources with little or no aggression" (Cronin and Sánchez, 2012, pp. 4).

Recent research has highlighted the importance of measuring and theorizing about the drivers of *intraspecific* variation in social tolerance. Cronin et al. (Cronin et al., 2014), for example, investigated the cofeeding tolerance levels of four groups of separately living sanctuary chimpanzees. The study utilized a group cofeeding paradigm in which a food resource (peanuts) was distributed within a predetermined feeding area. Cofeeding tolerance was then operationalized as the proportion of the group present in the feeding zone over the course of 2 minutes. The study found large inter-group differences in the proportion of the group able and motivated to forage in close proximity to each other, a pattern that was reflected in additional measures of sociality (Cronin et al., 2014). This finding is consistent with previous research on differences in sociality in wild populations. For example, two studies have assessed social tolerance in wild chimpanzees with a wide array of different measures (e.g. time spent in parties, meat sharing, female grooming, and medicinal plant use) and found a consistent pattern of cross-site differences that corresponded to differences in socially learned skills (van Schaik et al., 1999; van Schaik, 2003).

Researchers have also found differences in social behavior between groups of chimpanzees within the same field site. For example, two communities at Kibale have shown differences in female gregariousness (Watts, 2012) and clique formation (Wakefield, 2013), which has been proposed to be (partially) the result of reduced feeding competition and increased group-size (Wakefield, 2013). More general inter-community differences in sociality among males have also been found between the South and East Groups of Taï, with differences in aggressive and cooperative behaviors and some measures of general gregariousness being reported, possibly resulting from differing levels of within- and between-group competition, as well as demographic differences (Preis et al., 2019b). These two groups have also been shown to display cultural differences in a wide array of behaviors ranging from tool use to hunting behavior (Luncz and Boesch, 2015). The two communities of chimpanzees at Budongo—Sonso

and Waibira—have also shown differences in their meat sharing behavior (Hobaiter et al., 2017), the reasons for which remain to be determined. These findings, combined with those on captive populations, demonstrate that groups of chimpanzees can be characterized by different social styles and that these differences can be observed even when the groups are living under comparable ecological conditions (Cronin et al., 2014).

An important outstanding question, however, pertains to the temporal stability of such intraspecific, cross-group differences in social styles. Longitudinal study of group-specific social tolerance would provide important information about the possible mechanisms by which intraspecific variation in sociality emerges: possibly by group-specific (cultural) interaction styles (e.g., van Leeuwen et al., 2018), or by more transient individual- or demography-dependent phenomena, or by some combination of both.

Certain aspects of chimpanzee sociality are known to be temporally stable; for example, dyadic relationships can be maintained over many years (Gilby and Wrangham, 2008; Kossi et al., 2012; Langergraber et al., 2009; Rosati et al., 2020), as can some alpha male tenures (Goodall, 1986). It is unclear, however, to which extent this stability extends to group-level social styles. In one of the few studies on temporal change in primate social styles, Sapolsky and Share (Sapolsky and Share, 2004) observed the *de novo* emergence and continuation of a socially tolerant and relaxed social climate (e.g., higher rates of grooming and affiliation) in a troop of wild baboons over a 20 year period. This change was instigated by the abrupt deaths of many of the troop's more aggressive males, resulting in a troop with an unusually high number of relatively peaceful males. The resultant peaceful climate persisted over generations (Sapolsky and Share, 2004). Another study reported short-term changes in sociability in the Kasekala chimpanzees between 1977 and 1979 (Goodall, 1986). These differences were attributed to the differing number of estrous females present in the group over time, as estrous females were shown to be more gregarious, and to attract more males than anestrous females. These reports provide provisional support for a certain level of temporal flexibility in primate social climates and suggest that they may be influenced by group demographics.

The current state of knowledge concerning the effect of demographic variables on chimpanzee sociality, and more specifically, social tolerance, however, is ambiguous. Male and female chimpanzees differ in their sociability with males being more gregarious and having more and stronger bonds to other males than females (Lonsdorf et al., 2014; Pepper et al., 1999; Wilson, 2012). These sex-based differences could lead to groups with a higher ratio of males to females being more socially cohesive and possibly more socially tolerant of one another. A low female-to-male ratio could also increase males' willingness to tolerate females as a mating strategy (Pruetz and Lindshield, 2012). On the other hand, more males and therefore fewer females in a group could increase scramble competition for access to females and therefore decrease males' tolerance of one another (Fawcett and Muhumuza, 2000).

The possible effect of females on group-level sociality is further complicated by their state of estrous. Being in estrous has been found to increase female chimpanzees' gregariousness (Pepper et al., 1999), with some researchers finding estrous females to be as gregarious as males (Matsumoto-Oda, 1999), which might lead them to also be more socially tolerant of other females and males. Estrous females also attract males (Hashimoto et al., 2001; Matsumoto-Oda, 1999), which can lead to an overall increase in sociability within a group (Goodall, 1986).

The number of infants and juveniles could also affect group-level sociability. On the one hand, the presence of an infant is likely to decrease its mothers' willingness to be in close proximity to other individuals—especially adult males—so as to avoid male aggression (Lowe et al., 2019; Otali and Gilchrist, 2006). On the other hand, infants and juveniles typically experience high levels of tolerance from adult chimpanzees (von Rohr et al., 2011). Infants and juveniles may also lead to increased social tolerance by providing play partners, as play behavior has been proposed to decrease stress and increase tolerance in feeding contexts among captive primates (Norscia and Palagi, 2011; Palagi et al., 2004). Hence, it is conceivable that the various demographic factors outlined above affect group-level social tolerance in opposing ways. For example, a large number of infants and juveniles in a group could decrease *cofeeding tolerance*, because the mothers, especially those with smaller infants, stay away from the feeding context, while, at the same time, increase *overall social tolerance* by providing opportunities for group members to relieve stress through play behavior.

Another aspect of chimpanzee sociality that was originally assumed to have a large influence on both male and female relationships is kinship (Goodall, 1986). Subsequent research, however, has found little robust support for the effect of kinship on association patterns (Goldberg and Wrangham, 1997; Langergraber et al., 2009; Lehmann

and Boesch, 2009; Mitani et al., 2000; cf. Surbeck et al., 2017), cooperation (Eppley et al., 2013; Langergraber et al., 2007; Mitani et al., 2000), or grooming behavior (Gomes et al., 2009; Rodrigues and Boeving, 2019; cf. Foerster et al., 2015; Lehmann et al., 2006). Taken together, these results suggest that kinship plays at most a limited role in group-level chimpanzee sociality.

Finally, larger group size appears to lead to the formation of cliques and less overall group cohesiveness (Lehmann and Boesch, 2004; Wakefield, 2013), but appears to have little effect on other aspects of group sociality among chimpanzees (Lehmann and Boesch, 2004). Research on social tolerance among Japanese macaques also found no effect of group size on social tolerance (Kaigaishi et al., 2019). On the other hand—as mentioned above—research on social tolerance among non-primates has found a positive relationship between group size and social tolerance (D'Eath and Keeling, 2003; Dardenne et al., 2013).

In the current study, we investigate whether there is temporal variability in group-specific social tolerance using the same cofeeding tolerance assay (referred to as the *peanut swing*, see Figure S1 and Video S1 for more details on the experimental setup) in the same chimpanzee groups as those described in Cronin et al. (Cronin et al., 2014), but now longitudinally over the course of 8 years. Given the lack of precedents, we approach this study in an exploratory manner. Our primary aims are to: (1) identify the extent of variability of cofeeding tolerance (a) between the groups, and (b) within the groups over time. Furthermore, based on previous research demonstrating the importance of demographic variables, we investigate: (2) whether group demographics are associated with group-level cofeeding tolerance as measured by our experimental assay. Specifically, we explore the associations of cofeeding tolerance with: (a) the number of females vs. males, (b) the number of kin vs. non-kin, as well as (c) the fraction of individuals belonging to different age groups (e.g., infants, juveniles, adolescents, and adults). Due to the exploratory nature of this study, and the lack of consistent findings in the extant literature, we do not formulate directed hypotheses concerning these variables.

Lastly, in the final year of data-collection, we introduced a group-level tolerance measure which employed a codrinking instead of a cofeeding paradigm (referred to as the *juice pipe* see Figure S1 and Video S2 for more details on the experimental setup) to cross-check the validity of our main assay by comparing the outcomes of both approaches.

The study took place at Chimfunshi Wildlife Orphanage Trust (Chimfunshi) in four neighboring groups of chimpanzees comprising approximately 100 individuals. See Figure 1 for geographic details and Table 1 for demographic details. For further information about these populations and relevant animal care protocols, see the Transparent Methods section "Study site and subjects".

Results

To model the cofeeding tolerance of our chimpanzee groups, we built a statistical model that explicitly follows the structure of the experimental setup. The experiment was repeated several times in each year, in each group. We thus estimate effects unique to the interaction of group and year. Measurements of cofeeding in each experimental session were taken at fixed time-points after the introduction of peanuts. We explicitly account for the effects of peanut depletion on inferred cofeeding tolerance with a dynamic consumption model within each experimental session (for more details see the Transparent Methods section "A statistical model for the peanut swing data generating process" and Figures S2-S7) .

Cofeeding Tolerance by Year and Group

Figure 2 plots the time series of maximal cofeeding tolerance by group. Maximal cofeeding tolerance can be understood as the model-estimated initial tolerance level—the proportion of the group present before any of the peanuts are consumed or carried away—which accounts for differential rates of resource depletion across the groups. We observe evidence of reliable differences in cofeeding tolerance both across chimpanzee groups within years, and evidence of reliable difference across years within groups. This provides evidence that cofeeding tolerance is not a temporally stable, group- or species-level property. However, the range of variation here does not span the full set of possible values, and so we cannot rule out the possibility of a species-specific range of cofeeding tolerance levels—with, for example, these groups of chimpanzees being more tolerant than a species with a different, consistently lower range of estimated tolerance values.

Variation in Cofeeding Tolerance across Years and Groups

We observe evidence of reliable changes in cofeeding tolerance across both years and groups in Figure 2. This variation is quantified in Transparent Methods Figure S8. We find that there is reliably greater across-group variation in cofeeding tolerance in some years (i.e., 2011 and 2012) than in other years (i.e., 2015 and 2016). There is also evidence that some groups (i.e., Group 1) show greater temporal variability in cofeeding tolerance than other groups (i.e., Group 2 or 4). Finally, we find evidence of greater inter-group differences within years than intra-group differences across years in maximal cofeeding tolerance (for more details see Transparent Methods section "Variation in cofeeding tolerance across years and groups").

Demographic Correlates of Cofeeding Tolerance

To assess the demographic predictors of the observed variation, both across groups as well as within groups over time, we used two models: a multi-level model with group-specific coefficient vectors (that reflect the within-group effects of covariates) and a standard model with a single coefficient vector shared across all groups (that can reflect the between-group effects of covariates). Figure 3 plots the results of both of these models, and illustrates the demographic predictors of maximal cofeeding tolerance. An increasing frequency of juvenile and adolescent female chimpanzees in the population is associated with increased cofeeding tolerance in both within-group and between-group models. An increasing frequency of young infants under three years of age is also associated with decreased cofeeding tolerance in both within-group and between-group models. Additional effects of group size and maternal kin frequency are apparent in between-group analyses.

Counter-Factual Predictive Simulations

To further investigate the extent to which cofeeding tolerance is explained by demographic variables, we ran predictive simulations of the time-series of maximal cofeeding tolerance by group, conditional on counter-factually removing variation in demographic variables. Counter-factually removing demographic variation across groups, we find that inter-group differences are attenuated, but not completely removed, implying some random effects of group outside of those attributable to differences in demographic composition: Group 1 remains the least tolerant and Group 2 the most tolerant group (see Transparent Methods Figures S9-S12). These simulation analyses demonstrate that demographic variation explains much of the intra-group variation—and some, but not all, of the inter-group variation—in maximal cofeeding tolerance. Further details about these and additional counter-factual predictive simulations can be found in Transparent Methods section "Counter-factual predictive simulations".

An Individual-Level Network Analysis of Dyadic Cofeeding Tolerance

For a subset of peanut swing sessions, we were able to code the identities of all individual chimpanzees present at the food zone at each scan ($n = 1,264$ individual observations). As a follow-up of our findings regarding the influence of group-level demographics on cofeeding tolerance (see section 1.3. Demographic correlates of cofeeding tolerance), we explored the propensities of individuals of specific sex/age categories to be in the food zone, expressed both in individual and dyadic terms ($n = 9,280$ dyadic observations). To account for repeated observations of individuals and dyads, we used `Stan` code from Pisor et al. (2019) to implement the Social Relations Model (Kenny and La Voie, 1984; Koster et al., 2020). See Transparent Methods section "The social relations model" for complete details on this model.

Our network analysis of coresidence in the food zone indicates that, at the individual level, females with infants under age 3 are somewhat less likely than other individuals to reside in the food zone across scans. At the dyadic level, females with infants under age 3 are less likely to coreside in the food zone with adult or adolescent males, while adult females without infants are more likely to coreside with adult males. See Figure 4 for additional discussion.

Generalizability of the Assay

Finally, we compared estimates of maximal cofeeding tolerance taken in 2018 using both the peanut swing and the juice pipe methodologies. We find that, under either assay, the rank order of groups by maximal cofeeding tolerance is identical (see Transparent Methods Figure S13). The cross-group differences, however, are starker under the juice pipe assay than the peanut swing. Moreover, the estimates are more precise under the juice pipe procedure.

Discussion

In this study, we measured the cofeeding tolerance levels of four groups of chimpanzees longitudinally over the course of eight years to explore whether cofeeding tolerance is a stable group-level characteristic and which demographic factors, if any, might influence group-level cofeeding tolerance levels over time. We find that cofeeding tolerance in these groups of chimpanzees is a highly flexible construct that displays an appreciable amount of variation over time. These changes do not represent random fluctuations, but correspond closely to changes in the demographic composition of each group.

The frequency of infants under three years of age had a negative within- and between-group influence on cofeeding tolerance. Our finer-scale social network analysis reveals that females with infants under three years of age were indeed somewhat less likely to be present in the food zone than females without young infants. Females with young infants were especially less likely to be in the food zone when adult and adolescent males were present. This is presumably due to the mothers of young infants refraining from being in close proximity to other individuals, especially males, so as to minimize aggressive behaviors directed towards themselves and their offspring (Lowe et al., 2019; Otali and Gilchrist, 2006). We expect this kind of situationally dependent cofeeding avoidance behavior to have a strong temporal component, decreasing as the infants grow older and are less vulnerable, and then increasing again with the birth of new, highly vulnerable infants.

In the literature, much of the male-female aggression observed in chimpanzees is assumed to be sexually motivated (i.e., selectively directed toward parous and maximally sexually swollen females) (Muller et al., 2007, 2009) [but see (Stumpf and Boesch, 2010)], and more common in adult than adolescent males (Muller et al., 2009). Non-sexually motivated male-female aggression, however, has been shown to be most frequent in adolescent and young adult males (Muller et al., 2009), and is hypothesized to be motivated by young males' attempts to climb the dominance hierarchy (Muller et al., 2009; Nishida, 2003). This could explain why females with small infants were most strongly deterred by adolescent males.

The frequency of juvenile and adolescent females within the population was found to have both within- and between-group effects. Groups with larger numbers of juvenile and adolescent females showed higher levels of cofeeding tolerance. This could possibly reflect an effect of females in estrous, who are known to show increased gregariousness (Matsumoto-Oda, 1999) and to also attract males (Hashimoto et al., 2001; Matsumoto-Oda, 1999). While we do not have sufficient data on the females' states of estrous over the eight years, our age categories of juveniles and adolescents likely contained females already in estrous (female chimpanzees are known to reach menarche earlier in captivity (Atsalis and Videan, 2009; Coe et al., 1979)). Since older females are more likely to either have a more permanent form of contraceptive or be pregnant or nursing (for more details on Chimfunshi's breeding policies, see the Transparent Methods section"Study site and subjects") the age categories of juveniles and adolescents may best account for cycling females without dependent young. Groups with higher numbers of juvenile and adolescent females might therefore have more females in estrous, increasing the overall number of individuals willing to be in close proximity while cofeeding. However, since this effect was not found at the individual level—i.e. adolescent and juvenile females were not more likely than females of other age categories to cofeed in our experiment, or to coreside with adult males—this interpretation should be treated with caution, and we suggest that future research further investigate the possible direct and indirect effects of female behavior on group-level cofeeding tolerance.

Strier et al. (Strier et al., 2014) identify two types of behavioral traits: constraining traits—which are temporally stable and respond slowly to change—and responding traits—which are temporally and locally variable. They found that grouping pattern (e.g., stable core or fission-fusion) was a responding trait among 22 primates species and highly dependent on demographic changes (Strier et al., 2014). Given the variation we have observed in chimpanzees' group-level cofeeding tolerance, our results suggest that the propensity to join in close proximity to others in the vicinity of depleting, valuable food resources is another such responding trait, reflecting the context-dependent nature of chimpanzees social behavior.

However, not all of the inter-group variation could be explained by demographic variables and there was reliably more variation between groups than across years. As such, our results also imply a certain amount of group-level stability in social tolerance levels. This group effect could reflect an additional influence of group-specific factors, such as learned behavioral styles (van Leeuwen et al., 2018) or the more emergent phenomenon of

so-called *collective/group personalities* (Wright et al., 2019; Bengston and Jandt, 2014). Group-level differences have been found for the personality trait *sociability* among chimpanzees (Koski, 2011) and may be affected by socio-environmental factors such as group size and key individuals (Wright et al., 2019; Bengston and Jandt, 2014; Koski and Burkart, 2015; Cronin et al., 2014).

Cronin et al. (Cronin et al., 2014) compared cofeeding tolerance with two additional measures: the evenness of the distribution of food resources (calculated with Pielou's measure of J (Pielou, 1977)) and the average association indices in the social group outside of an experimental context. In one cross-sectional study, these measures of sociality were associated with one another (Cronin et al., 2014). It remains unknown, however, how different measures of sociality compare in their temporal stability: some aspects of sociality might be temporally stable and others much more dynamic. A recent study by van Leeuwen et al. (van Leeuwen et al., 2018) investigated (non-food-related) spatial proximity, grooming proclivities, and party size among the four groups in Chimfunshi and found consistent differences in group-level sociality such that the groups with relatively high rates of spatial associations were also characterized by stronger grooming bonds and larger party sizes. These differences, however, displayed a high level of consistency over the course of the three years in which the data were gathered (2011-2013), suggesting that they could represent temporally stable differences in sociality at a group level (e.g., cultures). The aspects of sociality analyzed in van Leeuwen et al. (van Leeuwen et al., 2018) were based on individuals' choices of whom to associate with and groom, likely reflecting their relationships with group members, which are known to be relatively stable among chimpanzees (Gilby and Wrangham, 2008; Kossi et al., 2012; Langergraber et al., 2009). The cofeeding measure that is the focus of the current study, on the other hand, reflects the upper limit of individuals' willingness to be with conspecifics, in a competitive situation. It is possible that willingness to cofeed, as a responding trait, is a more flexible and transient behavioral characteristic than is dyadic bonding.

We also found group size to have a positive effect and the frequency of maternal kin to have a negative effect on cofeeding tolerance when comparing between groups. However, we did not find an effect of these predictors on changes in cofeeding tolerance over time within groups. This is due to the lower within-group variation as opposed to between-group variation in these variables in our data. It is possible that between-group differences in these variables are causally related to difference in cofeeding tolerance, but given our study design it is difficult to rule out confounding.

In our final year of data collection, we introduced a secondary measure of cofeeding tolerance, the juice pipe. Both assays reveal the same inter-group pattern of cofeeding tolerance levels, validating our previous measure, the peanut swing. The juice pipe was shown to be an improvement in measuring cofeeding tolerance, as it ensures a stable resource level within each session and reduces the variance in food zone size across sessions, resulting in more precise and reliable measurements of cofeeding tolerance. As such, the juice pipe represents an improved paradigm for future studies on cofeeding tolerance, when resources allow.

Social tolerance is often discussed as a species-specific trait and, as such, has been used to describe and characterize many different species. Our study, however, demonstrates that social tolerance levels within a species— as measured with a cofeeding paradigm—can vary substantially, both among groups as well as within groups over time. This said, our results do not preclude the possibility of a species-level component to social tolerance. The cofeeding tolerance levels we illustrate here do not span the full set of possible values, and so it is conceivable that the typical range of cofeeding tolerance values in chimpanzees differs from the typical ranges of other species, for example, bonobos (see Cronin et al., 2015). Species can also be characterized by different extents of variability, possibly reflecting different levels of behavioral flexibility (Kamilar and Baden, 2014). To address these possibilities, however, we would require data-set of measurements over multiple years from multiple groups. Our study highlights the need for future research to not only consider inter-group variation but also intra-group variation over time. A full comprehension of the breadth of intraspecific variation will allow us to better understand the extent to which social tolerance is a necessary precondition for successful group living.

Limitations of the Study

There is, to date, a lack of research focused on comparing and integrating different theories and operationalizations of social tolerance (see DeTroy et al., Manuscript submitted for publication), which limits the generalizations

that can be made from our findings on cofeeding tolerance in chimpanzees to other measures of social tolerance in other species. Future research would benefit from studies systematically comparing a variety of measures of social tolerance and sociability in multiple species with longitudinal data.

A further factor which could have influenced cofeeding tolerance in chimpanzees that we did not consider is rank-stability. There were three changes in alpha-male over the course of the eight years (in Groups 1, 2, and 3), with two additional ongoing challenges (in Groups 3 and 4) during our final year of measurement. While there are not enough instances of such rank changes to support formal statistical modeling, such social changes warrant further attention as rank-stability has been shown to affect sociability in wild and captive chimpanzees (Gilby and Wrangham, 2008; Hemelrijk and Ek, 1991; Koyama et al., 2017; Preis et al., 2019a).

Our results are also based on chimpanzees living with supplemented human care. While our study groups live in large outdoor enclosures, enabling individuals to display natural fission-fusion dynamics for the majority of the day (see van Leeuwen et al., 2018, 2019), they are provisioned twice a day. As a result—and contrary to the natural conditions of wild chimpanzees—the four groups experience a constant and stable level of resource availability. In wild settings, increased resource availability has been posited to have led to increased female sociability in a comparison of two neighboring wild chimpanzee populations (Wakefield, 2013) and to explain the differences in social tolerance between Sumatran and Bornean orangutans (Schuppli et al., 2017). Seasonal differences in resource availability have also been shown to be a good predictor for party size among wild chimpanzees (Fawcett, 2000). Research comparing cofeeding tolerance in wild and captive redfronted and ringtailed lemurs have found captive populations of both species to have higher levels of cofeeding tolerance, a result attributed to higher food availability in captive populations (Fichtel et al., 2018). On the other hand, among Japanese macaques, provisioning has been observed to decrease social tolerance, presumably because it causes group members to gather in competitive situations more often than they would under natural conditions (Kaigaishi et al., 2019; Hill, 1999).

Our data show that while resource availability may play a role in cofeeding tolerance, it is not the sole influencer, as we observe substantial cross-group variation in cofeeding tolerance even when resource availability is held experimentally fixed. Similar findings have been shown in studies of wild chimpanzees (Lehmann and Boesch, 2004). By working with multiple chimpanzee groups in the same captive context, we can investigate the effects of lesser-studied sources of variation in cofeeding tolerance—such as the demographic composition of the group—without our estimates being confounded by differences in resource access. Future research would benefit from investigating how the effects of resource availability interact with the effects of demographic variables.

Resource Availability

Lead Contact

Further information and requests for resources and reagents should be directed to and will be fulfilled by the Lead Contact, Sarah E. DeTroy (sarah_detroy@eva.mpg.de).

Materials Availability

This study did not generate new unique reagents.

Data and Code Availability

Code and data for diagnostics and analysis replication are available at the Open Science Framework repository https://osf.io/meq59/?view_only=3cda5b91921a4178b6286955da16538c.

Acknowledgments

We wish to thank the Max Planck Institute for Evolutionary Anthropology and the University of Leipzig for funding this research. E.J.C.v.L. was additionally funded by the Research Foundation Flanders (FWO) - 12W5318N. We thank Innocent Mulenga (general manager) for facilitating the research; Thalita Calvi (on-site veterinarian) for her research-supporting efforts; the Zambian research team, including Patrick Chambatu,

Thomson Mbilishi, Albert Mulembo, Goodson Muletele, Felix Chinyama, Patrick Mwika, Mumba Kawele, Misheck Kasongo, John Kayuya, Richard Kafunga, Rodgers Chilombo, Robert Sokontwe, Ndema Sakala, and Joseph Kasongo; Mark Bodamer and the Gonazaga University students for continued research support; all the peanut swingers, especially Anni Gläser, Clara Dubois, Manon Schweinfurth and Bianca Dietrich; Dirk Diemer, Daniel DeTroy and Sebastian Schütte for engineering advice; Amelie Conrad and Mackenzie Hewes for coding support; Anne Sibilsky, Maleen Thiele, Sarah Peoples, and Linda Schymanski for help with the Graphical Abstract; the Zambia Wildlife Authority; the Chimfunshi Board of Trustees; and the Chimfunshi Research Advisory Board.

Author Contributions

S.E.D., E.J.C.v.L., and D.B.M.H designed research; S.E.D, E.J.C.v.L., and K.A.C. performed research; C.T.R. analysed data, S.E.D., E.J.C.v.L., C.T.R., K.A.C., and D.B.M.H. wrote this paper. All authors gave final approval for publication.

Declaration of Interest

The authors declare no competing interests.

References

Amici, F., Call, J., Aureli, F., 2012. Aversion to violation of expectations of food distribution: the role of social tolerance and relative dominance in seven primate species. Behaviour 149, 345–368.

Atsalis, S., Videan, E., 2009. Reproductive aging in captive and wild common chimpanzees: factors influencing the rate of follicular depletion. American Journal of Primatology 71, 271–282.

Balasubramaniam, K.N., Beisner, B.A., Berman, C.M., De Marco, A., Duboscq, J., Koirala, S., Majolo, B., MacIntosh, A.J., McFarland, R., Molesti, S., et al., 2018. The influence of phylogeny, social style, and sociodemographic factors on macaque social network structure. American Journal of Primatology 80, e22727.

Balasubramaniam, K.N., Dittmar, K., Berman, C.M., Butovskaya, M., Cooper, M.A., Majolo, B., Ogawa, H., Schino, G., Thierry, B., de Waal, F.B.M., 2012. Hierarchical steepness, counter-aggression, and macaque social style scale. American Journal of Primatology 74, 915–925.

Bengston, S.E., Jandt, J.M., 2014. The development of collective personality: the ontogenetic drivers of behavioral variation across groups. Frontiers in Ecology and Evolution 2, 1–13.

Bonanni, R., Cafazzo, S., Abis, A., Barillari, E., Valsecchi, P., Natoli, E., 2017. Age-graded dominance hierarchies and social tolerance in packs of free-ranging dogs. Behavioral Ecology 28, 1004–1020.

Bradshaw, J.W.S., 2016. Sociality in cats: a comparative review. Journal of Veterinary Behavior 11, 113–124.

Burkart, J.M., Allon, O., Amici, F., Fichtel, C., Finkenwirth, C., Heschl, A., Huber, J., Isler, K., Kosonen, Z.K., Martins, E., et al., 2014. The evolutionary origin of human hyper-cooperation. Nature Communications 5, 4747.

Burkart, J.M., Hrdy, S.B., van Schaik, C.P., 2009. Cooperative breeding and human cognitive evolution. Evolutionary Anthropology 18, 175–186.

Burkart, J.M., van Schaik, C., 2013. Group service in macaques (*Macaca fuscata*), capuchins (*Cebus apella*) and marmosets (*Callithrix jacchus*): a comparative approach to identifying proactive prosocial motivations. Journal of Comparative Psychology 127, 212–226.

Calcutt, S.E., Lonsdorf, E.V., Bonnie, K.E., Milstein, M.S., Ross, S.R., 2014. Captive chimpanzees share diminishing resources. Behaviour 151, 1967–1982.

Chudek, M., Henrich, J., 2011. Culture–gene coevolution, norm-psychology and the emergence of human prosociality. Trends in Cognitive Sciences 15, 218–226.

Cieri, R.L., Churchill, S.E., Franciscus, R.G., Tan, J., Hare, B., 2014. Craniofacial feminization, social tolerance, and the origins of behavioral modernity. Current Anthropology 55, 419–443.

Clay, Z., de Waal, F.B.M., 2013. Development of socio-emotional competence in bonobos. Proceedings of the National Academy of Sciences 110, 18121–18126.

Coe, C.L., Connolly, A.C., Kraemer, H.C., Levine, S., 1979. Reproductive development and behavior of captive female chimpanzees. Primates 20, 571–582.

Cronin, K.A., 2012. Prosocial behaviour in animals: the influence of social relationships, communication and rewards. Animal Behaviour 84, 1085–1093.

Cronin, K.A., 2017. Comparative studies of cooperation: collaboration and prosocial behavior in animals., in: Call, J., Burghardt, G.M., Pepperberg, I.M., Snowdon, C.T., Zentall, T. (Eds.), APA Handbook of Comparative Psychology: Basic concepts, methods, neural substrate, and behavior, Vol. 1. American Psychological Association, Washington, DC, pp. 925–929.

Cronin, K.A., De Groot, E., Stevens, J.M.G., 2015. Bonobos show limited social tolerance in a group setting: a comparison with chimpanzees and a test of the relational model. Folia Primatologica 86, 164–177.

Cronin, K.A., van Leeuwen, E.J.C., Vreeman, V., Haun, D.B.M., 2014. Population-level variability in the social climates of four chimpanzee societies. Evolution and Human Behavior 35, 389–396.

Cronin, K.A., Sánchez, A., 2012. Social dynamics and cooperation: the case of nonhuman primates and its implications for human behavior. Advances in Complex Systems 15, 1250066.

Dardenne, S., Ducatez, S., Cote, J., Poncin, P., Stevens, V.M., 2013. Neophobia and social tolerance are related to breeding group size in a semi-colonial bird. Behavioral Ecology and Sociobiology 67, 1317–1327.

D'Eath, R.B., Keeling, L.J., 2003. Social discrimination and aggression by laying hens in large groups: from peck orders to social tolerance. Applied Animal Behaviour Science 84, 197–212.

DeTroy, S.E., Haun, D.B.M., van Leeuwen, E.J.C., Manuscript submitted for publication. What isn't social tolerance? The past, present, and possible future of an overused term in the field of primatology .

Duboscq, J., Micheletta, J., Agil, M., Hodges, K., Thierry, B., Engelhardt, A., 2013. Social tolerance in wild female crested macaques (*Macaca nigra*) in Tangkoko-Batuangus Nature Reserve, Sulawesi, Indonesia. American Journal of Primatology 75, 361–375.

Eppley, T.M., Suchak, M., Crick, J., de Waal, F.B.M., 2013. Perseverance and food sharing among closely affiliated female chimpanzees. Primates 54, 319–324.

Fawcett, K., Muhumuza, G., 2000. Death of a wild chimpanzee community member: possible outcome of intense sexual competition. American Journal of Primatology 51, 243–247.

Fawcett, K.A., 2000. Female relationships and food availability in a forest community of chimpanzees. Ph.D. thesis. University of Edinburgh. Edinburgh.

Fehr, E., Fischbacher, U., 2003. The nature of human altruism. Nature 425, 785–791.

Fichtel, C., Schnoell, A.V., Kappeler, P.M., 2018. Measuring social tolerance: an experimental approach in two lemurid primates. Ethology 124, 65–73.

Foerster, S., McLellan, K., Schroepfer-Walker, K., Murray, C.M., Krupenye, C., Gilby, I.C., Pusey, A.E., 2015. Social bonds in the dispersing sex: partner preferences among adult female chimpanzees. Animal Behaviour 105, 139–152.

Forss, S.I.F., Willems, E., Call, J., van Schaik, C.P., 2016. Cognitive differences between orang-utan species: a test of the cultural intelligence hypothesis. Scientific Reports 6, 30516.

Fruth, B., Hohmann, G., 2018. Food sharing across borders. Human Nature 29, 91–103.

Fuentes, A., 2004. It's not all sex and violence: integrated anthropology and the role of cooperation and social complexity in human evolution. American Anthropologist 106, 710–718.

Ganem, G., Bennett, N.C., 2004. Tolerance to unfamiliar conspecifics varies with social organization in female African mole-rats. Physiology & Behavior 82, 555–562.

Gilby, I.C., Wrangham, R.W., 2008. Association patterns among wild chimpanzees (*Pan troglodytes schweinfurthii*) reflect sex differences in cooperation. Behavioral Ecology and Sociobiology 62, 1831–1842.

Goldberg, T.L., Wrangham, R.W., 1997. Genetic correlates of social behaviour in wild chimpanzees: evidence from mitochondrial dna. Animal Behaviour 54, 559–570.

Gomes, C.M., Mundry, R., Boesch, C., 2009. Long-term reciprocation of grooming in wild West African chimpanzees. Proceedings of the Royal Society B 276, 699–706.

Goodall, J., 1986. The chimpanzees of Gombe: Patterns of behavior. Harvard University Press, Cambridge.

Hare, B., 2017. Survival of the friendliest: *Homo sapiens* evolved via selection for prosociality. Annual Review of Psychology 68, 155–186.

Hare, B., Melis, A.P., Woods, V., Hastings, S., Wrangham, R., 2007. Tolerance allows bonobos to outperform chimpanzees on a cooperative task. Current Biology 17, 619–623.

Hare, B., Wobber, V., Wrangham, R., 2012. The self-domestication hypothesis: evolution of bonobo psychology is due to selection against aggression. Animal Behaviour 83, 573–585.

Hashimoto, C., Furuichi, T., Tashiro, Y., 2001. What factors affect the size of chimpanzee parties in the Kalinzu Forest, Uganda? examination of fruit abundance and number of estrous females. International Journal of Primatology 22, 947–959.

Hemelrijk, C.K., Ek, A., 1991. Reciprocity and interchange of grooming and 'support' in captive chimpanzees. Animal Behaviour 41, 923–935.

Hill, D.A., 1999. Effects of provisioning on the social behaviour of Japanese and rhesus macaques: implications for socioecology. Primates 40, 187–198.

Hobaiter, C., Samuni, L., Mullins, C., Akankwasa, W.J., Zuberbühler, K., 2017. Variation in hunting behaviour in neighbouring chimpanzee communities in the Budongo forest, Uganda. PLoS ONE 12, e0178065.

Jaeggi, A.V., Stevens, J.M.G., van Schaik, C.P., 2010. Tolerant food sharing and reciprocity is precluded by despotism among bonobos but not chimpanzees. American Journal of Physical Anthropology 143, 41–51.

Kaigaishi, Y., Nakamichi, M., Yamada, K., 2019. High but not low tolerance populations of Japanese macaques solve a novel cooperative task. Primates 60, 421–430.

Kamilar, J.M., Baden, A.L., 2014. What drives flexibility in primate social organization? Behavioral Ecology and Sociobiology 68, 1677–1692.

Kenny, D.A., La Voie, L., 1984. The social relations model, in: Advances in experimental social psychology. Elsevier. volume 18, pp. 141–182.

Koski, S.E., 2011. Social personality traits in chimpanzees: temporal stability and structure of behaviourally assessed personality traits in three captive populations. Behavioral Ecology and Sociobiology 65, 2161–2174.

Koski, S.E., Burkart, J.M., 2015. Common marmosets show social plasticity and group-level similarity in personality. Scientific Reports 5, 8878.

Kossi, S.E., de Vries, H., van de Kraats, A., Sterck, E.H.M., 2012. Stability and change of social relationship quality in captive chimpanzees (*Pan troglodytes*). International Journal of Primatology 33, 905–921.

Koster, J., Leckie, G., Aven, B., 2020. Statistical methods and software for the multilevel social relations model. Field Methods 32, 339–345.

Koyama, N.F., Ronkainen, K., Aureli, F., 2017. Durability and flexibility of chimpanzee grooming patterns during a period of dominance instability. American Journal of Primatology 79, e22713.

Langergraber, K., Mitani, J., Vigilant, L., 2009. Kinship and social bonds in female chimpanzees (*Pan troglodytes*). American Journal of Primatology 71, 840–851.

Langergraber, K.E., Mitani, J.C., Vigilant, L., 2007. The limited impact of kinship on cooperation in wild chimpanzees. Proceedings of the National Academy of Sciences 104, 7786–7790.

Lee, N.S., Goodwin, N.L., Freitas, K.E., Beery, A.K., 2019. Affiliation, aggression, and selectivity of peer relationships in meadow and prairie voles. Frontiers in Behavioral Neuroscience 13, 52.

van Leeuwen, E.J.C., Cronin, K.A., Haun, D.B.M., 2018. Population-specific social dynamics in chimpanzees. Proceedings of the National Academy of Sciences 115, 11393–11400.

van Leeuwen, E.J.C., Cronin, K.A., Haun, D.B.M., 2019. Reply to Farine and Aplin: chimpanzees choose their association and interaction partners. Proceedings of the National Academy of Sciences 116, 16676–16677.

Lehmann, J., Boesch, C., 2004. To fission or to fusion: effects of community size on wild chimpanzee (*Pan troglodytes verus*) social organisation. Behavioral Ecology and Sociobiology 56, 207–216.

Lehmann, J., Boesch, C., 2009. Sociality of the dispersing sex: the nature of social bonds in West African female chimpanzees, *Pan troglodytes*. Animal Behaviour 77, 377–387.

Lehmann, J., Fickenscher, G., Boesch, C., 2006. Kin biased investment in wild chimpanzees. Behaviour 143, 931–955.

Lonsdorf, E.V., Anderson, K.E., Stanton, M.A., Shender, M., Heintz, M.R., Goodall, J., Murray, C.M., 2014. Boys will be boys: sex differences in wild infant chimpanzee social interactions. Animal Behaviour 88, 79–83.

Lowe, A.E., Hobaiter, C., Newton-Fisher, N.E., 2019. Countering infanticide: chimpanzee mothers are sensitive to the relative risks posed by males on differing rank trajectories. American Journal of Physical Anthropology 168, 3–9.

Luncz, L.V., Boesch, C., 2015. The extent of cultural variation between adjacent chimpanzee (*Pan troglodytes verus*) communities; a microecological approach. American Journal of Physical Anthropology 156, 67–75.

Matsumoto-Oda, A., 1999. Mahale chimpanzees: grouping patterns and cycling females. American Journal of Primatology 47, 197–207.

McShea, W.J., 1990. Social tolerance and proximate mechanisms of dispersal among winter groups of meadow voles, *Microtus pennsylvanicus*. Animal Behaviour 39, 346–351.

Melis, A.P., Hare, B., Tomasello, M., 2006. Engineering cooperation in chimpanzees: tolerance constraints on cooperation. Animal Behaviour 72, 275–286.

Miller, R., Schiestl, M., Whiten, A., Schwab, C., Bugnyar, T., 2014. Tolerance and social facilitation in the foraging behaviour of free-ranging crows (*Corvus corone corone; C. c. cornix*). Ethology 120, 1248–1255.

Mitani, J.C., Merriwether, D.A., Zhang, C., 2000. Male affiliation, cooperation and kinship in wild chimpanzees. Animal Behaviour 59, 885–893.

Muller, M.N., Kahlenberg, S.M., Emery Thompson, M., Wrangham, R.W., 2007. Male coercion and the costs of promiscuous mating for female chimpanzees. Proceedings of the Royal Society B 274, 1009–1014.

Muller, M.N., Kahlenberg, S.M., Wrangham, R.W., 2009. Male aggression against females and sexual coercion in chimpanzees, in: Muller, M., Wrangham, R. (Eds.), Sexual Coercion in Primates and Humans. Harvard University Press Cambridge, London, pp. 184–217.

Nishida, T., 2003. Harassment of mature female chimpanzees by young males in the Mahale Mountains. International Journal of Primatology 24, 503–514.

Norscia, I., Palagi, E., 2011. When play is a family business: adult play, hierarchy, and possible stress reduction in common marmosets. Primates 52, 101–104.

Otali, E., Gilchrist, J.S., 2006. Why chimpanzee (*Pan troglodytes schweinfurthii*) mothers are less gregarious than nonmothers and males: the infant safety hypothesis. Behavioral Ecology and Sociobiology 59, 561–570.

Palagi, E., Cordoni, G., Borgognini Tarli, S., 2004. Immediate and delayed benefits of play behaviour: new evidence from chimpanzees (*Pan troglodytes*). Ethology 110, 949–962.

Pepper, J.W., Mitani, J.C., Watts, D.P., 1999. General gregariousness and specific social preferences among wild chimpanzees. International Journal of Primatology 20, 613–632.

Petit, O., Desportes, C., Thierry, B., 1992. Differential probability of "coproduction" in two species of macaque (*Macaca tonkeana, M. mulatta*). Ethology 90, 107–120.

Pielou, E.C., 1977. Mathematical Ecology. Wiley, New York.

Pisor, A.C., Gervais, M.M., Purzycki, B.G., Ross, C.T., 2019. Preferences and constraints: the value of economic games for studying human behaviour. Royal Society Open Science 7, 192090.

Pisor, A.C., Surbeck, M., 2019. The evolution of intergroup tolerance in nonhuman primates and humans. Evolutionary Anthropology 28, 210–223.

Preis, A., Liran, S., Deschner, T., Crockford, C., Wittig, R.M., 2019a. Urinary cortisol, aggression, dominance and competition in wild, West African male chimpanzees. Frontiers in Ecology and Evolution 7, 107.

Preis, A., Samuni, L., Deschner, T., Crockford, C., Wittig, R., 2019b. Group-specific social dynamics affect urinary oxytocin levels in Taï male chimpanzees, in: Boesch, C., Wittig, R.M. (Eds.), The Chimpanzees of the Taï Forest: 40 Years of Research. Cambridge University Press, Cambridge, pp. 339–365.

Pruetz, J.D., Lindshield, S., 2012. Plant-food and tool transfer among savanna chimpanzees at Fongoli, Senegal. Primates 53, 133–145.

Richerson, P., Baldini, R., Bell, A.V., Demps, K., Frost, K., Hillis, V., Mathew, S., Newton, E.K., Naar, N., Newson, L., et al., 2016. Cultural group selection plays an essential role in explaining human cooperation: a sketch of the evidence. Behavioral and Brain Sciences 39.

Rodrigues, M.A., Boeving, E.R., 2019. Comparative social grooming networks in captive chimpanzees and bonobos. Primates 60, 191–202.

von Rohr, C.R., Burkart, J.M., van Schaik, C.P., 2011. Evolutionary precursors of social norms in chimpanzees: a new approach. Biology & Philosophy 26, 1–30.

Rosati, A.G., Hagberg, L., Enigk, D.K., Otali, E., Emery Thompson, M., Muller, M.N., Wrangham, R.W., Machanda, Z.P., 2020. Social selectivity in aging wild chimpanzees. Science 370, 473–476.

Sapolsky, R.M., Share, L.J., 2004. A pacific culture among wild baboons: its emergence and transmission. PLoS Biology 2, 294–304.

van Schaik, 2003. Local traditions in orangutans and chimpanzees: social learning and social tolerance, in: Fragaszy, D., Perry, S. (Eds.), The biology of traditions: Models and evidence. Cambridge University Press, New York, pp. 297–328.

van Schaik, C.P., Deaner, R.O., Merrill, M.Y., 1999. The conditions for tool use in primates: implications for the evolution of material culture. Journal of Human Evolution 36, 719–741.

Schuppli, C., Forss, S., Meulman, E., Atmoko, S.U., van Noordwijk, M., van Schaik, C., 2017. The effects of sociability on exploratory tendency and innovation repertoires in wild Sumatran and Bornean orangutans. Scientific Reports 7, 15464.

Strier, K.B., Lee, P.C., Ives, A.R., 2014. Behavioral flexibility and the evolution of primate social states. PloS ONE 9, e114099.

Stumpf, R.M., Boesch, C., 2010. Male aggression and sexual coercion in wild West African chimpanzees, *Pan troglodytes verus*. Animal Behaviour 79, 333–342.

Surbeck, M., Girard-Buttoz, C., Boesch, C., Crockford, C., Fruth, B., Hohmann, G., Langergraber, K.E., Zuberbühler, K., Wittig, R.M., Mundry, R., 2017. Sex-specific association patterns in bonobos and chimpanzees reflect species differences in cooperation. Royal Society Open Science 4, 161081.

Tan, J., Hare, B., 2013. Bonobos share with strangers. PLoS ONE 8, e51922.

Thierry, B., 2007. Unity in diversity: lessons from macaque societies. Evolutionary Anthropology 16, 224–238.

de Waal, F.B.M., 1986. Class structure in a rhesus monkey group: the interplay between dominance and tolerance. Animal Behaviour 34, 1033–1040.

Wakefield, M.L., 2013. Social dynamics among females and their influence on social structure in an East African chimpanzee community. Animal Behaviour 85, 1303–1313.

Watts, D.P., 2012. Long-term research on chimpanzee behavioral ecology in Kibale National Park, Uganda, in: Kappeler, P., Watts, D. (Eds.), Long-term field studies of primates. Springer, Berlin, pp. 313–338.

Wild, S., Hoppitt, W.J.E., Allen, S.J., Krützen, M., 2020. Integrating genetic, environmental, and social networks to reveal transmission pathways of a dolphin foraging innovation. Current Biology 30, 3024–3030.

Wilson, M.L., 2012. Long-term studies of the chimpanzees of Gombe National Park, Tanzania, in: Kappeler, P., Watts, D. (Eds.), Long-term field studies of primates. Springer, Berlin, pp. 357–384.

Wright, C.M., Lichtenstein, J.L.L., Doering, G.N., Pretorius, J., Meunier, J., Pruitt, J.N., 2019. Collective personalities: present knowledge and new frontiers. Behavioral Ecology and Sociobiology 73, 31.

Figure Captions

Figure 1. Aerial View of the Enclosures. Aerial view of the four enclosures at Chimfunshi (modified from Google Maps).

Figure 2. Time-Series of Cofeeding Tolerance in Four Groups of Chimpanzees. Each bar plots the central 90 percent credible interval of maximal cofeeding tolerance (i.e., initial cofeeding tolerance) in each group of chimpanzees in each year. Bars are jittered around year for visual clarity, but all empirical observations were matched in time. We observe substantial variation in cofeeding tolerance, both across groups, and within groups across years. See also Figures S1-S6 and S8-S13, Tables S3-S4, and Video S1.

Figure 3. Density Plots of Covariates. Density plots of the effects of various covariates on maximal cofeeding tolerance, with central 90% credible intervals in blue. The group-specific plots show the within-group effects of a change in a covariate on the maximal cofeeding tolerance in that group (i.e., all parameters are random effects by group). The overall plots (the bottom plot in each panel) show the between-group effects (i.e., when the same parameters are shared across all groups). Note that for the covariates "Infants (<3 years)" and "Females (5-12 years)" very little of the central 90% credible intervals overlap with 0, indicating reliable within- as well as between-group effects. For the parameters "Group Size" and "Maternal Kin" this is only the case for the between-group effects. See also Figure S8.

Figure 4. Estimates from the Social Relations Model. Each bar plots the central 90 percent credible interval of the regression coefficients giving the change in log-odds of a cofeeding tie as a function of the indicated variable. Bars with little or no overlap with 0 indicate reliable positive or negative effects of the respective variable. Bars are clustered by age category on the left axis, with color showing unique estimates by sex within each age category and shading indicating sample size. In the top frame, we plot individual-level random effects estimates for the interaction of sex and age category on residence in the food zone. In the bottom frame, we plot random effects estimates for dyads composed of females with or without infants under 3 years old (indicated by color) and males of various age categories (indicated by rows). Adult females with infants under age 3 and young females are less likely to coreside in the food zone with adult or adolescent males, while adult females without infants under age 3 are more likely to coreside with adult males. Females, with or without infants, are more likely to coreside with males ages 5 to 8 than with males of other age classes.

Table Captions

Table 1. Demographic Overview. The range (min–max) of the number of individuals, the percentage of females, and the percentage of individuals with maternal kin, in each of the four groups, 2011-2018. See also Table S1.

Supplemental Methods Videos

Video S1. A Peanut-Swing Session in Group 2, 2017. Related to Figure 2.

Video S2. A Juice-Pipe Session in Group 2, 2018. Additional video associated with SI Figure 13. Note that due to the time lag between juice coming out of the holes at the beginning of the pipe and at the end of the pipe, the session doesn't properly start until 00:40.
